# Nanostructured Zirconia-Based Ceramics and Composites in Dentistry: A State-of-the-Art Review

**DOI:** 10.3390/nano9101393

**Published:** 2019-09-29

**Authors:** Antonio Arena, Francesca Prete, Elisa Rambaldi, Maria Chiara Bignozzi, Carlo Monaco, Adolfo Di Fiore, Jérôme Chevalier

**Affiliations:** 1 Department of Biomedical and Neuromotor Sciences, University of Bologna, 40125 Bologna, Italy; carlo.monaco2@unibo.it; 2 Centro Ceramico, 40138 Bologna, Italy; prete@centroceramico.it (F.P.); rambaldi@centroceramico.it (E.R.); maria.bignozzi@unibo.it (M.C.B.); 3 Department of Civil, Chemical, Environmental and Materials Engineering, University of Bologna, 40131 Bologna, Italy; 4 Department of Neurosciences, University of Padua, 35133 Padua, Italy; adolfo.difiore@unipd.it; 5 Materials Science Department, University of Lyon, INSA Lyon, CNRS UMR 5510 MATEIS, 69621 Villeurbanne, France; jerome.chevalier@insa-lyon.fr

**Keywords:** nano ZrO_2_, nano Y-TZP, TZP/Al_2_O_3_ nanocomposite, nano dental restorations, nano dentistry

## Abstract

The objective of this paper is to review the current knowledge on the development of nanostructured zirconia-based ceramics and composites suitable for application in dentistry. Isi Web of Science, Science Direct, Scientific.net databases, and Google were searched electronically for the period of 1980 to the present, matching the keywords “nano” with the keywords: “Zirconia, ZrO_2_, Y-TZP, and dental, dentistry”. A total of 74 papers were found, with the majority coming from Asia, indicating a more active scientific interest on the topic in this geographic area, followed by Europe, South America, and North America. The research shows, even though the scientific activity on nanostructured ceramics was intense in the last fifteen years, the development of fully dense zirconia-based nanoceramics is yet at an initial stage, most of all from the point of view of the clinical applications. It has been demonstrated that nanostructured ceramics can show improved properties because of the reduction of the grain size to the nanoscale. This is also true for zirconia-based nanoceramics, where some improvements in mechanical, optical, as well as resistance in low-temperature degradation have been observed. Potential applications of this class of material in the dental field are discussed, summarizing the results of the latest scientific research.

## 1. Introduction

In this review, a literature search was conducted on the databases Isi Web of Science, Science Direct, Scientific.net, and Google Scholar, for the period of 1980 to the present, matching the keyword “nano”, the keywords zirconia, ZrO_2_, Y-TZP, and the keywords dental and dentistry. A total of 74 papers that met these requirements were found.

For the purpose of this research, some preliminary considerations about the literature references and the definition of nanostructured materials need to be made. Following the definition proposed by Gleiter [1], nanostructured materials are solids composed of structural elements with a characteristic size (at least in one direction) of a few nanometer, typically from 1 to 100 nm.

Furthermore, it has been observed that, in scientific literature, zirconia-based ceramic materials with microstructural elements (grain size, GS) greater than 100 nm (sometimes GS equal to 500–600 nm) are also often defined as “nano”.

Very few studies were successful in obtaining fully dense nanostructure-based zirconia ceramics (GS < 100 nm, T.D. ≈ 99%) since, as illustrated in this review, it is very difficult to retain the microstructure on a real nanometric level.

**Figure 1:**
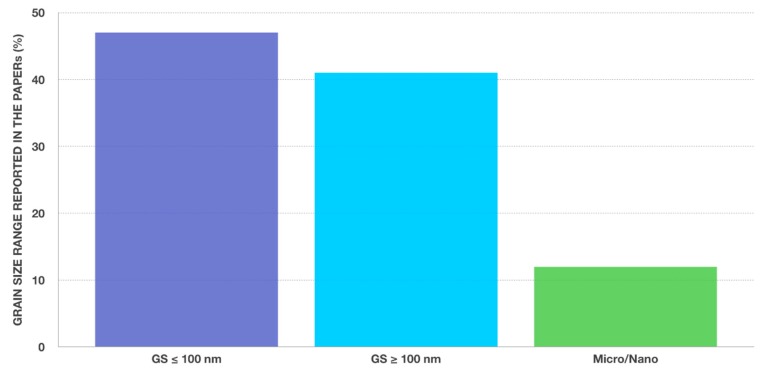
Percentage of scientific papers concerning the development of nanostructured zirconia-based ceramics on the basis of the grain size (GS) of the developed products.

**Figure 2:**
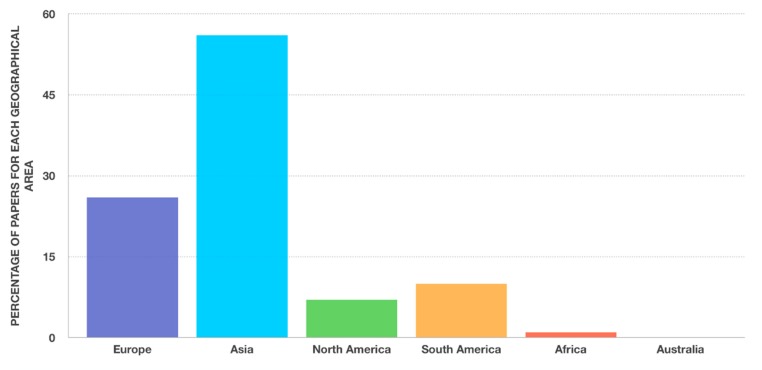
Geographical provenience of scientific papers on nanostructured-based zirconia components found in this research.

Figure 1 reports a subdivision of the papers on the basis of the grain size of the developed final products: For the elaboration of Figure 1, only the paper reporting an experimental procedure to produce nanozirconia-made components were considered. In Figure 2, the geographical provenience of all the 74 analyzed scientific papers [1,2,3,4,5,6,7,8,9,10,11,12,13,14,15,16,17,18,19,20,21,22,23,24,25,26,27,28,29,30,31,32,33,34,35,36,37,38,39,40,41,42,43,44,45,46,47,48,49,50,51,52,53,54,55,56,57,58,59,60,61,62,63,64,65,66,67,68,69,70,71,72,73,74] is reported. The subdivision has been made taking into account the affiliation of the first author of each paper with the aim to identify which geographic area is working more intensively on this topic.

Ceramic materials have been used for a long time in dentistry for the production of dental restorations due to their excellent biocompatibility, their good mechanical properties, and their aesthetic appearance, very similar to that of natural teeth.

In the last 15 years, the technological evolution behind the development of this class of materials has been remarkable and their use is becoming even more intensive [75], due to the growing request of patients around the world asking for metal free solutions. The development of totally metal free solutions is one of the main research topics in the field of dental materials and, even though metal alloys are still extensively used, their unnatural color and the insurgence of unwanted chemical–biological interactions connected with their use is encouraging the use of ceramics even more. At now, among the new research trends, there is the development of zirconia-based ceramics and composites having a nanometric structure, since several studies have demonstrated that nanostructured materials’ properties are different and generally improved, in respect to that of conventional materials.

A literature review of Cain and Morrell [76] summarizes the benefits connected to the use of nanostructured ceramics: The reduction of the grain size leads to an improvement of the strength, since finer grain size leads to stronger materials, the firing temperature can be reduced saving energy due to shorter firing times and lower temperatures, optical properties can be changed and the translucency can be improved resulting in the opportunity of producing transparent or more translucent ceramics. Nanostructured advanced ceramic materials have already been used in neurosurgery to manufacture prototypes of transparent calvarium prosthesis with the aim of avoiding repeated craniectomies after surgery [77]. The use of nanostructured alumina in the form of fibers and spheres has been proposed as a material for orthopedic implants with the aim of reproducing the structure of natural bone with a biomimetic approach [78]. The application of nanostructured zirconia and ceramic nanocomposites as material to manufacture hip prosthesis seem to greatly reduce the friction and wear and to increase stability, reliability, and durability of the prosthesis as a whole [79,80]. Despite these applications in the biomedical field, to date, little has been written on ceramic materials with nanoscale structure in dentistry [81]. This review covers the existing scientific literature on nanostructured zirconia-based ceramics and composites suitable for application in dentistry and turns the attention also to the existing commercial products.

## 2. Shaping and Sintering of Nanostructured Zirconia-Based Ceramics

The development of nanostructured ceramics implies, theoretically, a revision of the traditional shaping and sintering processes, with respect to conventional micrometric ceramic materials. On the nanometer scale, the surface properties start becoming more relevant than the bulk material properties, since a great part of the atoms is located on the surface of the initial particles and on the surface and boundaries of the grains in the final product.

The very small size of the starting powder particles, which are characterized by a great chemical reactivity, promotes lower sintering temperature and lower sintering times, which could ideally lead to increased production rates at reduced costs. The same high reactivity is, however, responsible for the agglomeration of nanoparticles that causes the emergence of large pores with inhomogeneous particle distribution leading to low density in the green compacts and in the final products.

The larger the agglomerate size, the higher the sintering temperature required to obtain fully dense materials [19]. A high sintering temperature, however, causes grain growth, leading the final product to became micrometric once the full density is reached. For this reason, to retain the microstructure at a nanometric level and, at the same time, avoiding the grain coarsening, is a bottle neck encountered in the processing of this class of materials.

Another major problem is related to the number of particle–particle point contacts per unit volume in a nanocrystalline powder that is much higher compared to a micrometric powder. Therefore, the frictional resistance to compaction is more pronounced.

Despite the described difficulties, several studies have demonstrated the possibility to develop nanostructured-based zirconia components suitable for dental applications. It is important to point out that, even though the research activity on ceramic nanomaterials was intense in the last 14 years and zirconia nanopowders are widely commercially available, only a very few studies were successful in developing fully dense zirconia-based products with grain size below or not too far from 100 nm. Some of these studies [10,16,17,27,37,41,45,46,52,66,67] are based on the use of conventional or adapted shaping and sintering techniques, such as slip casting, uniaxial pressing, cold isostatic pressing, powder injection molding, hot isostatic, pressing followed by conventional sintering or two step sintering. The two step sintering method, in particular, originally developed by Chen and Wang [82], seems to be specifically suitable for the densification of nanostructured ceramics since it limits the grain growth. This kind of sintering comprises a first step carried out at a higher temperature for a short period of time and then a rapid cooling to complete the second step at a lower temperature, which is maintained for a prolonged time. In this second step occurs the elimination of pores in a frozen microstructure with suppression of grain growth.

Other studies [2,3,5,6,25,26,41] are, instead, based on new and rapid sintering techniques, such as microwave sintering (characterized by many advantages compared to a conventional sintering process such as lower temperatures, shorter times, energy saving) and spark plasma sintering that have shown to produce nanocrystalline ceramics with relative high densities.

Beyond these studies, there are many others, including in their title or in the abstract of the word “nano”, but referring to the development of zirconia-based products (also including zirconia composites) with grain size generally much higher than 100 nm [9,12,13,14,18,20,21,22,24,34,40,49,51,53,54,57,60,61,65,68], following conventional processing routes or innovative routes, such as one of the latest innovations in shaping techniques, such as the magnetic pulsed compaction [8], or reporting no information on the final grain size [7,11,12,55].

Other studies report the development of micro/nano zirconia-based composites suitable for dental applications [30,31,32,47,56,57,59,71].

## 3. Properties of Zirconia at the Nanoscale

### 3.1. Limiting Low-Temperature Degradation (LTD) Kinetics in Y-TZP

Zirconia-based ceramics are widely known in dentistry thanks to the combination of their mechanical properties [83]. The optimum aesthetical appearance and the excellent biocompatibility and are widely used for the realization of endodontic posts, crown, bridges, and implant abutments. However, some zirconia-based ceramics suffer from an important drawback, which is their sensitivity to Low-Temperature Degradation (LTD), also known as hydrothermal aging [84]. This phenomenon, which has been extensively documented and well explained in previous reports [85,86,87,88,89], raises serious limitations to the applications of these materials in the biomedical field, especially in the oral environment, where zirconia-based ceramics are exposed to oral fluids and mechanical stress, causing possible irreversible premature failure of zirconia components.

Several studies have demonstrated that there is a relationship between the LTD resistance and the grain size and that a microstructure composed of nanometric grains leads to a suppression or to a reduction of the LTD sensitivity [90,91,92,93]. Nanostructured fully dense zirconia-based ceramics resistant to hydrothermal aging, with a grain size of <100 nm, were developed by Paul et al. [10] and with a grain size of <200 nm by Matsui et al. [14]. Transparent 3Y-TZP nanoceramics with an average grain size of 87 nm that show no noticeable low-temperature degradation after 100 h aging at 134 °C under a hydrothermal pressure of two bars were developed by Xiong at al. [15]. Fully dense materials with a critical grain size of < 0.36 μm (not “nano” in *sensustricto*) that do not show any evidence of degradation after extreme aging conditions at pressurized autoclaving in hot water at 100, 200, and 260 °C for 8 h were developed by Munoz-Saldana et al. [92]. This result was also reported by Hallmann et al. [93] that confirm that an average grain size of the dental Y-TZP below 0.3 μm is able to inhibit the phase transition under LTD conditions or at least decrease the transformation kinetics to negligible amount during the product lifetime.

### 3.2. Avoiding LTD by the Development of Composites with Alternative Dopants

A ceramic nanocomposite (NANOZR) was launched at the end of 90 s by Panasonic Electric Works. NANOZR was constituted by a matrix of 10 mol% CeO_2_ stabilized TZP and 30 vol% Al_2_O_3_ as a second phase. The nanostructure was designed so that many 10–100 nm Al_2_O_3_ particles were trapped within the ZrO_2_ grains, and that many 10 nm ZrO_2_ particles were trapped within the Al_2_O_3_ grains [62].

Tanaka et al. [31] found, after hydrothermal aging, only a slight variation in the content of the monoclinic phase in a Ce-TZP/Al nanocomposite while in the conventional Y-TZP such content reached 25.3%. Ban et al. [55] have performed an aging test of 14 days at 120 °C on Y-TZP and NANOZR. The authors reported an increase in the content of the monoclinic phase (from 3.8% to 75.5%) only for Y-TZP. In addition, after aging, no reduction in biaxial flexural strength of NANOZR was found while that of Y-TZP decreased by 17%.

Other authors [59] assessed the in vivo (implantation into rabbit tibiae) and in vitro (autoclaving or storage in physiological saline solution) effects of aging on stability of the tetragonal phase of Ce-TZP/Al_2_O_3_ nanocomposite and Y-TZP. Ce-TZP/Al_2_O_3_ nanocomposite showed a remarkable stability of the tetragonal phase, no decrease in strength, and no alteration of surface quality. On the contrary, the content of the monoclinic phase of Y-TZP was close to 80%. These results were confirmed by Perdigao et al. [35].

The effect of LTD on mechanical properties of ceramic nanocomposites was evaluated by comparing the biaxial flexural strength and the content of monoclinic zirconia inside NANOZR and a conventional Y-TZP before and after aging. The content of the monoclinic phase inside NANOZR was almost unchanged (from 4.8 vol% to 5.5 vol%) while in the conventional Y-TZP the increase was much more pronounced (from 0.3 vol% to 49.9 vol%). Similarly, the biaxial flexural strength of NANOZR was minimally reduced (from 1422 MPa to 1371 MPa), while that of conventional Y-TZP was decreased (from 1046 MPa to 892 MPa) [30].

Sim et al. [94] reported that occlusal adjustments performed using a superfine diamond bur can cause an increase in the t-m transformation and that Ce-TZP/Al_2_O_3_ had a higher flexural strength and greater resistance to LTD than Y-TZP.

### 3.3. Optical Properties

Reproducing a dental restoration that satisfactorily mimics the natural appearance of the teeth is one of the main challenges in dentistry: The appearance of a dental restoration depends on its optical properties, on the illumination condition, and on the optical properties of the core substructure. Among the different optical properties, an adequate translucency is essential to reproduce a realistic dental restoration. Translucency is the relative amount of light transmitted through a material [95] and is related to the thickness of the ceramic layer [96] and is strongly dependent on light scattering [97]. A translucent material allows some light to pass through it, absorbs some of the remainder, and scatters and reflects the rest from its surface or internal surface [98]. The scattering is due to several factors: The chemical nature, crystalline content, voids and porosity, and quantity and size of the crystals compared to the incident light wavelength [95]. An increase in crystalline content, while achieving greater strength, generally results in greater opacity [99,100]. For this reason, the polycrystalline ceramics, which are characterized by intense scattering effect, assume an opaque appearance [101] and are less translucent than glass ceramics. The clinical factors affecting the translucency of Y-TZP ceramics include: Thickness, cementation type, color of the monolithic zirconia, surface finishing methods and wear, dental background, cement color, and low-temperature degradation [102].

Casolco et al. [26] have recently shown that the translucency of partially stabilized zirconia can be improved significantly limiting, in the densely sintered material, the final size of crystals to 55 nm. This phenomenon, which has not yet been fully explained, is probably due to the fact that crystals of size smaller than the wavelength of visible light (400–700 nm) should not significantly hinder the passage of light [97]. Wang et al. [51] evaluated the influence of four heating rates (100°Kh^−1^, 200°Kh^−1^, 400°Kh^−1^, and 600°Kh^−1^) on the mechanical and optical properties of a nano structured yttria-stabilized zirconia. The authors found a correlation between the three higher heating rates (600°Kh^−1^, 400°Kh^−1^, 200°Kh^−1^) and the increase of the optical properties while no differences between the groups with regard to the mechanical properties were found. Jiang et al. [40] evaluated the effects of sintering temperature and particle size on the translucency of Y-TZP dental ceramics: The specimens were sintered at 1350 °C, 1400 °C, 1450 °C, and 1500 °C, and it was observed that the sintered densities and transmittances increased with the temperature from 1350 °C to 1500 °C.

Other studies [101] have evaluated the influence of the addition of alumina on the relative density and on the optical properties of zirconia/alumina nanocomposites. The increase in the percentage of the alumina from 0 to 10% causes a progressive decline in theoretical density and in transmittance of the composite ceramic material. Shiraishi et al. [101] reported that NANOZR becomes extremely opaque when this ceramic material is thicker than 0.3 mm. Pure zirconia nanopowders sintered densely could obtain the relatively high transmittance, while the transmittance and the lightness of slight addition of alumina changed significantly. The alumina addition influences the densification of the composite leading to a porosity increase that indirectly leads to a decreasing of the transmittance. Pores are highly efficient light scatters and thus very low porosity is required for ceramic to be transparent. The addition of alumina has an indirect effect on the transmittance of the composite ceramics through the porosity and the size of crystals. Zhang et al. [102] showed that it is possible to add small amounts of alumina or lanthanum oxide at the grain boundary that play a key role to decrease the LTD kinetics without compromising translucency.

### 3.4. Mechanical Properties

Zirconia-based ceramics exhibit the best mechanical properties ever attained among other ceramic materials. This is particularly true for TZP ceramics for which flexural strength ranges from 900 to 1200 MPa and fracture toughness from 7 to 10 MPa∙m^1/2^ [103,104,105].

For TZP ceramics, the high values of fracture toughness is related to the stress induced phase transformation from tetragonal to monoclinic, also known as, transformation toughening [106,107,108,109,110] that is accompanied by a volume increase on the order of 3–5% of grains, that generates compressive strength around the crack tip, thus, preventing the propagation of the crack. Traini et al. [111,112] showed that fracture toughness of Y-TZP was affected by surface grinding and that micrometric zirconia can be used for implant-supported restorations thanks to the high fracture strength value.

Due to the reduced grain size, nanostructured ceramics are expected to exhibit different and generally improved mechanical properties compared to conventional ceramic materials [41]. A microstructure with fine grain size, an excellent microstructural homogeneity and a fully dense structure is generally believed to generate improved mechanical properties.

Several studies have demonstrated an enhancement in the mechanical properties of nanostructured-based zirconia ceramics with respect to their micrometric counterparts. Dos Santos et al. [20] evaluated the properties of nanostructured 3Y-TZP blocks (ZrHP-nano^®^, see Section 5) used for CAD/CAM dental restorations and found that nanometric blocks (GS = 200 nm) had a hardness of 13 GPa, a fracture toughness of 11 MPa∙m^1/2^, a bending strength of 1020 MPa, and a Weibull modulus of 14, while micrometric ZrO_2_ blocks (GS = 500 nm) presented similar values of hardness, a fracture toughness of 8.5 MPa∙m^1/2^, a bending strength of 850 MPa, and a Weibull modulus of 10. Silva et al. [12] developed nanostructured yttria-stabilized zirconia blocks for dental applications and characterized them in terms of densification, crystalline phases, microstructure, and mechanical properties. They sintered the nanostructured blocks at 1400 °C (GS ≈ 250 nm) and the microstructured blocks (GS ≈ 1 μm) at 1600 °C and found a increase in flexural strength values (1020 MPa for nano and 855 MPa for micro), Weibull Modulus (13.1 for nano and 9.8 for micro), and fracture toughness values (11.2 MPa∙m^1/2^ for nano and 9.0 MPa∙m^1/2^). The higher fracture toughness was attributed to the maximization of volumetric fraction of retained tetragonal zirconia particles while the higher value of bending strength to induced nucleation of microcracks, increase of energy absorption during crack propagation, and developed compressive surface stress.

Other studies reporting some improvements on mechanical properties refer to the “nano/nano type” [46,67] or to the “micro/nano type” [9,56,62].

The Ce-TZP/Al_2_O_3_ nanocomposite, ascribing to the “micro/nano type” NANOZR^®^ possesses a very interesting combination of mechanical properties due to a particular microstructure [62] (see Section 5). A flexural strength of 1500 MPa, a fracture toughness of 18 MPa∙m^1/2^, an elastic modulus of 245 MPa, and a hardness of 12 GPa in addition to a very high resistance to LTD was found for this material.

Vasylkiv et al. [46] reported the production, by a colloidal technique and low-temperature sintering, of 0.75 to 3 mol% Y_2_O_3_ stabilized tetragonal zirconia and Al_2_O_3_/Y-TZP nanocomposites with 0.2 to 0.7 wt% of alumina, and determined the fracture toughness and hardness of the obtained samples. The bulk 2.7 Y-TZP with an average grain size of 110 nm reached a hardness of 13.6 GPa and a fracture toughness of 11.2 MPa∙m^1/2^ while the nanograined Al_2_O_3_/Y-TZP with an average size of 92 nm reached a hardness of 16.8 GPa. Y-TZP ceramics with a reduced yttria-stabilizer content were shown to reach a fracture toughness of 13.8 MPa∙m^1/2^ (2Y-TZP) and 14.5 MPa∙m^1/2^ (1.5Y-TZP). Y-TZP alumina composites with 0.35 wt% of alumina were shown to reach a fracture toughness of 15.7 MPa∙m^1/2^ (2Y) and 15.3 MPa∙m^1/2^ (1.5Y).

Another important mechanical property that has been observed for nanostructured ceramics is the superplasticity, that is the ability to deform in a ductile manner. This property, that make these material optimum candidates for near-net shape forming, has been so far observed for fine grained ceramics, not properly “nano” (with a grain size of <1 μm) that exhibit remarkable elongation to failure at moderate temperatures, typically half the melting point [19]. By further reducing the grain size to less than 100 nm it is possible to achieve much faster forming rates at much lower temperatures. The mechanism of deformation in nanosize ceramics occurs either by dislocation motion or by grain boundary sliding [39]. In nanoceramics of a grain size above ~100 nm, the main deformation mechanism is derived by dislocation motion, while for ultra-fine nano grain sizes (<50 nm) the deformation mechanism is derived by grain boundary sliding. YSZ exhibits superplastic strain rate 34 times faster than submicrometer grained YSZ when measured at the same temperature [36]. However, these properties, that have been demonstrated for several zirconia-based ceramics [47], seem to be not only dependent on the grain size: Domínguez-Rodríguez et al. [113], through a critical analysis of the plasticity of two important nanostructured oxide systems, MgO and yttria tetragonal zirconia polycrystals, show how nano-structuring may be a necessary, but not a sufficient condition for superplasticity in ceramics. A complex combination of effects and relationships are shown to modify the superplastic response.

## 4. Commercial Nanostructured Zirconia-Based Ceramics

An online research reveals that only two commercial products for dental application are defined as nanostructured: ZrHP-nano^®^ (ProtMatMateriaisAvançados^®^-Brazil) and NANOZR^®^ (Panasonic Healthcare–The Netherlands). Their main characteristics are reported in Table 1.

In the scientific literature, few papers (all from Brazil) refer to ZrHP-nano, but more papers deal with NANOZR.

ZrHP-nano is composed of 3 mol% of Y_2_O_3_ stabilized TZP and the average grain size is 150 nm, with crystallite dimensions of 18–70 nm [97].

NANOZR is composed of 10 mol% of CeO_2_ stabilized TZP as a matrix and 30 vol% of Al_2_O_3_ as second phase. The average size of the NANOZR is 0.49 μm. The significant characteristic of its structure is an intra-granular-type of nanostructure in which several 10–100 nm sized Al_2_O_3_ particles are trapped within the ZrO_2_ grains, and several 10 nm sized ZrO_2_ particles are trapped within the Al_2_O_3_ grains.

Han et al. [114] showed that NANOZR implants were biocompatible and capable of establishing a close bone–implant contact. A recent in vitro study performed by Komasa et al. [115] found that the modification of the NANOZR implant surface by alkali treatment can improve hard tissue formation surrounding implants. Rizo-Gorrita et al. [116,117] evaluated the early response of human gingival fibroblasts in contact with different materials and found that ceramic materials revealed a better cell response than the polymers. These results were consistent with findings from Okabe et al. [118]. This author found that NANOZR may impart resistance to exogenous stimuli through strong intercellular contacts with peri-implant mucosal cells when used as an abutment.

## 5. Clinical Trials and Indications of Nanostructured Zirconia-Based Ceramics

Up to now there are only two clinical trials on the use of nanostructured zirconia-based ceramics in dentistry. Philipp et al. [119] have reported promising results at one year of three unit-FPDs manufactured using a Ce-TZP/A-nanocomposite (NANOZR) as framework material. After a year of function, no failure of the framework and no chipping or fracture of the veneering ceramic was observed.

Tanaka et al. [120] assessed the clinical performance of veneered Ce-TZP/A-nanocomposite (three year follow-up) frameworks for fixed dental prostheses. Fifteen patients with 22 Ce-TZP/A-nanocomposite fixed prostheses were included in the study. Clinical events, including fracture and loss of retention, secondary caries, and marginal integrity, were recorded. The biologic outcome was judged by comparing the pre-treatment and post-treatment bleeding on probing (BOP) and probing pocket depth (PPD) of the abutment teeth. Radiographic examination was also performed at 12, 24, and 36 months. At 36 months of observation, the new Ce-TZP/A-nanocomposite prostheses exhibited a survival rate of 95.5%. Therefore, the new framework material was evaluated to be clinically reliable.

NANOZR can be used in a wide range of applications, including single crowns, long-span bridge frames, and fixed implant-supported prostheses.

Several authors [121,122] suggested designing a frame with a thickness of 0.3 mm on occlusal and axial surfaces. FPD’s connectors should be at least 3 mm × 3.5 mm. The framework should be veneered with a proper veneering ceramic. The material is not recommended to make veneers due to the low translucency (compared to dental glass-ceramics). In addition, these restorations cannot be adhesively luted since polycrystalline ceramics are not etchable.

Tanaka et al. [120] suggested to perform a sandblasting treatment, to apply a primer or a silane coupling agent on the material surface, and then to use a resin cement containing MDP (10-methacryloyloxy-decyldihydrogen-phosphate). This cementation protocol can be used also for micrometric zirconia [123]. The tooth should be prepared with a circumferentially rounded shoulder (width 0.8–1.0 mm), an occlusal reduction of 1.5–2.0 mm, and a proper conicity (6–10°).

## 6. Conclusions

Nanostructured ceramics can show improved properties because of the reduction of the grain size to the nanoscale. This is also true for zirconia-based nanoceramics, where these improvements can be used to develop highly reliable and aesthetic dental restorations. At present, the scientific research on nanostructured based zirconia components for dentistry has yet to overcome some important steps. One of the main goals is to retain the microstructure at the nanometric size and, at the same time, preserving the full density of the components, since very few studies were successful in obtaining fully dense nanostructure-based zirconia ceramics with a grain size of <100 nm.

Despite this difficulty, an interesting and alternative solution could be the use of micro/nano composites as, for example, Ce-TZP based nano-composites, that show optimum mechanical properties and good aging stability.

## Figures and Tables

**Table 1:** Synoptic table of the main characteristics of commercial nanostructured zirconia-based ceramics for dental applications.

Commercial Name	ZrHP-nano^®^	NANOZR^®^
Composition	Y-TZP	Ce-TZP and Al_2_O_3_
Average grain size, nm	150–240	490 matrix 10–100 precipitates
Density, r, g/cm^3^	6.05	5.56
Vickers hardness, HV, GPa	11–13	12
Fracture toughness, K_IC_, MPa√m	8–11	9–18
Flexural strength, s, MPa	1020	1422–1500
Elastic modulus, E, GPa	205	245
Weibull modulus, m	13–14	23
